# Eye Position Shifts Body Sway Under Foot Dominance Bias in the Absence of Visual Feedback

**DOI:** 10.3389/fneur.2022.835450

**Published:** 2022-03-30

**Authors:** Yoshiki Tamaru, Akiyoshi Matsugi

**Affiliations:** Faculty of Rehabilitation, Shijonawate Gakuen University, Daito, Japan

**Keywords:** eye position, body sway, postural control, dominant foot, visual reference, electrooculography

## Abstract

**Purpose:**

The purpose of this study was to investigate whether information on extraocular muscle proprioception without visual information affects postural control.

**Methods:**

Thirty-five healthy young volunteers participated in the study. Postural control outcomes included the center of pressure (CoP) for static standing, the total length of the sway of the CoP (LNG), and the sway area (SA), as well as the mean CoP in the mediolateral and anteroposterior directions. The following five eye-fixing positions were used: eye-up (E-Up), eye-down (E-Down), eye-right (E-Right), eye-left (E-Left), and eye-center (Center eye position). One-way ANOVA and Bonferroni correction was performed for statistical processing. Electrooculograms were recorded to detect eye orientation errors, measured with the eyes closed.

**Results:**

The results of this study showed no significant difference between the LNG and SA results when comparing respective eye positions (E-up, E-down, E-right, E-left) relative to E-Center (control). However, the average CoP was shifted to the right at E-Up, E-Down, and E-Left.

**Conclusion:**

These findings indicate that postural control may be affected by eye-body coordination depending on the position of the eyes, even without visual information.

## Introduction

Visual information contributes to postural stability in humans (1). Closing one's eyes increases body sway (2), and the change in optical flow in the peripheral visual field induces displacement of the foot center of pressure (CoP) during upright standing (3). Human vision captures targets through saccadic movements. The position of the retinal image is different before and after the saccade. This retinal displacement also affects postural instability (4). Gaze position (5, 6) and distance (7–9) are also factors that affect body sway. In terms of gaze direction, Paulus et al. (9) found that back-and-forth sway was induced by changes in target disparity over a short distance, while longitudinal sway was induced by the movement of the gaze following an object. In terms of gaze distance, Kapoula and Lê (6) and Moraes et al. (8) reported that postural sway in the left-right and front-back directions in healthy subjects was smaller when they gazed at a closer object than when they gazed at an object further away. In addition, eye movement accompanied by changes in visual reference affects postural control in humans (10). The lateral rhythmic movement of the eyes while following a moving target induces body sway in the mediolateral (ML) direction without inducing head movement (11). As these previous studies have shown, factors, such as eye position and focusing distance, along with visual information, affect postural control when the eyes are open. On the other hand, it is not clear whether changes in eye position alone affect body sway in the absence of external stimuli, such as when the eyes are closed. There is a substantial increase in body sway when the eyes are closed (9). In other words, the position of the eyes during eye closure is considered to affect the body sway. However, the way in which visual stimuli are integrated into postural control is not fully understood, and much research is needed to clarify the dynamic relationship between visual information and motor behavior (8). This interdependence of perception and action is thought to arise from the so-called action-perception cycle (12, 13).

The foot CoP is often used to assess body sway during upright standing. To determine the magnitude of body sway, the total length of the sway trajectory of the CoP (LNG) (14) and the area of the surface surrounded by CoP (SA) (15) have been used in previous studies. To determine the deviation in posture, the mean position of the CoP in the ML and anteroposterior (AP) directions is often used (14). As mentioned in these previous studies, it is well known that visual information contributes to postural control, in such a way that we can manipulate visual information with balance exercises. However, if not only visual information but also eye position itself influences postural control, then balance training programs should be updated to consider both visual information and eye position manipulation, for higher effectiveness. In the present study, we aimed to clarify whether proprioceptive information coming from the external ocular muscles involved in eye position affects body sway in the absence of visual information during the eyes-closed state.

## Materials and Methods

### Participants

In this study, the appropriate sample size was estimated with the G^*^power software (Version 3.1.9.4) (16) for one-way analysis of variance (ANOVA) with repeated measures for LNG and mean CoP to compare between eyes-directions. The type of power analysis was set to “A priori: Compute required sample size- given α, power, and effect size.” Effect size (f) was set to 0.5 (middle level), the α-error probability was set to 0.05, power (1-β-error probability) was set to 0.8, and correlation among repetitive measures was set to 0.5. The calculated sample size was 35. Therefore, 35 healthy subjects were recruited (male, 18; female, 17; mean age, 22.3 ± 2.4 years) in this study. All participants were right-footed, defined as habitually kicking a ball with the right foot (17). None of the participants had a history of neurological diseases. All participants were informed of the aim of the study and provided signed informed consent before participation, following the guidelines approved by the Shijonawate Gakuen University Faculty of Rehabilitation research ethics committee (Approval No. 18-10), and this study was conducted in accordance with the tenets of the Declaration of Helsinki.

### Experimental Procedure

**Figure 1:**
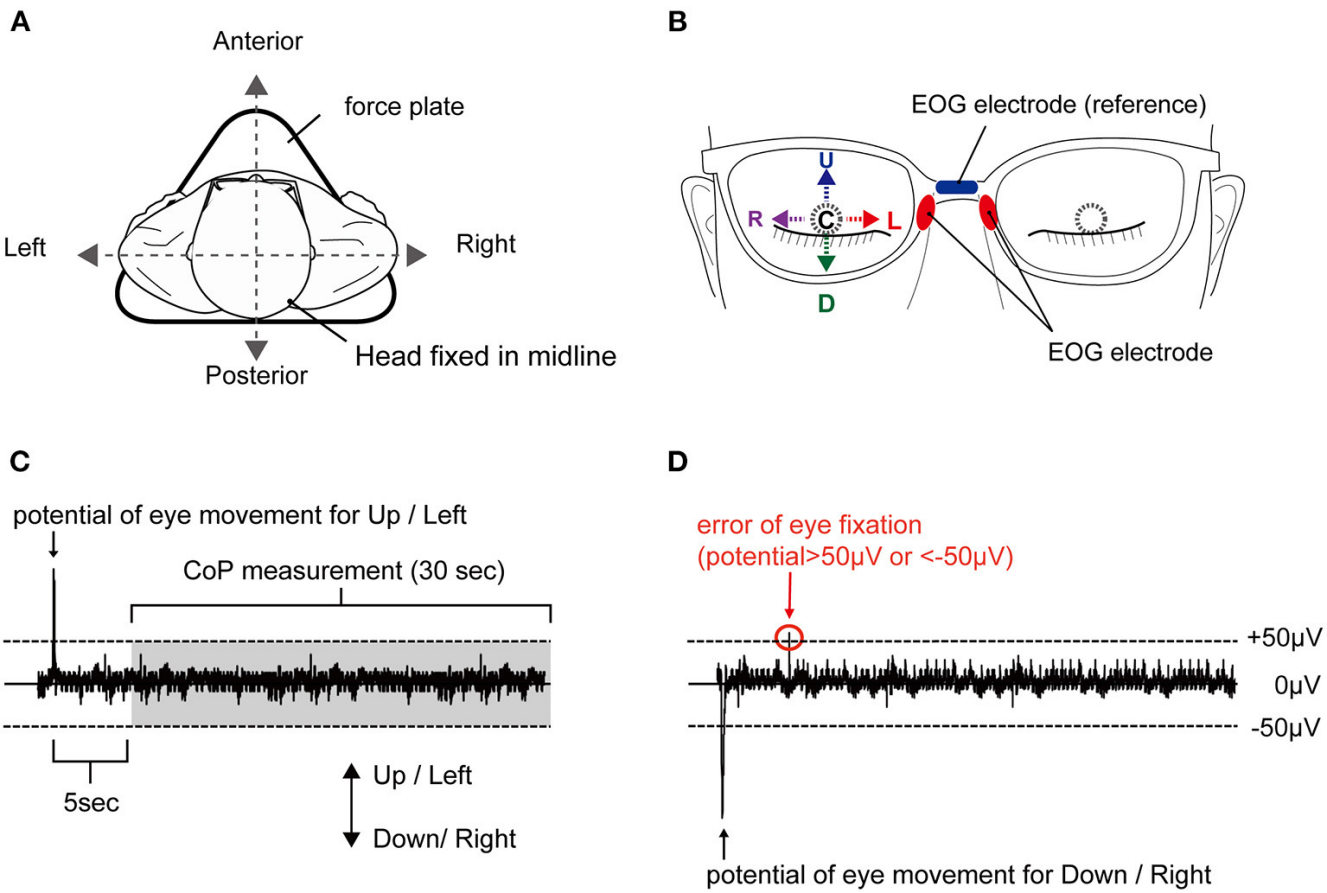
Experimental setup and typical EOG waveform. **(A)** Body sway was measured in a static standing position on a force plate, with no head movement. **(B)** Eye positions corresponding to E-Up (U), E-Down (D), E-Right (R), E-Left (L), and E-Center (C). **(C)** Appropriate waveform and **(D)** inappropriate waveform. A plus sign reflects a shift to the anterior, and a minus sign reflects a shift to the posterior. The waveform is considered inappropriate with an EOG of ±50 μV or higher in a direction different from the target direction. EOG, electrooculography.

The participants were asked to stand motionless on a footprint that was pre-printed on a force plate, with both toes of the first digit of the feet pointed outward at an angle of 15°, with heel contact and eyes closed during all experiments (see Figure 1A). Next, participants were instructed to fix their eyes in five positions: E-Up, E-Down, E-Right, E-Left, E-Center (see Figure 1B). The eye position was initiated by an external cue. The participants were asked to maintain their eye position for 60 s without inducing body sway or head movements. We conducted the randomization of the task order regarding eye direction using Microsoft Excel software for Mac (version 16.16.10; Microsoft Corp., Redmond, WA, USA). Trials were conducted at 180-s intervals. One attempt was made for each position. Electrooculography (EOG) of the right eye was performed and monitored online during the task, while the CoP measurement began after approximately 5 s into the 30-s holding of the eye position (see Figure 1C). In the EOG waveforms of this study, E-Up and E-Right were shown as positive waveforms, and E-Down and E-Right were shown as negative waveforms. In each trial, subjects fixed their eyes in the position specified by an external stimulus from the examiner, which was confirmed by the appearance of the EOG waveform. If the EOG value, which reflects the position of the eye in the horizontal and vertical directions, exceeded ±50 μV in a different direction from that of the attended direction, the trial was reconducted (see Figure 1D).

### EOG and CoP Measurements

EOG was used to confirm the direction of movement of the right eye with eyes closed, without contaminating visual information. EOG was assessed as described previously (18) using JINS MEME EOG glasses (JINS Inc., Tokyo, Japan) (19). The dry electrodes of the ocular potential sensors were placed on the left and right nose pads, and the reference electrode was placed on the upper part of the nose pad (see Figure 1B). The sampling frequency was set to 100 Hz. The device has an EOG sensor that can measure in the X and Y-axes. The EOG data were simultaneously transferred from this EOG system to a smartphone device using Bluetooth during the experiment. The data were also transferred to a computer via the ES_R Development Kit application (JINS Inc., Tokyo, Japan), as in a previous study (18). To estimate the deviation of the body from that in the no-movement eye task, the CoP of each participant's foot while standing was measured using a force plate (Gravicorder G5500; Anima, Tokyo, Japan). The sampling rate was set to 20 Hz. To estimate body sway, the LNG and SA were calculated. To estimate the deviation of the CoP, the mean CoP position in the AP and ML directions was assessed.

### Statistical Analysis

The Shapiro–Wilk test was used to confirm that the data had a normal distribution. We conducted the parametric statistical analysis, as normality was confirmed. One-way ANOVA was used to compare whether the difference in eye position affects the mean in the AP and ML directions and the body sway (LNG, SA). If there was an effect of eye position, *post-hoc* Bonferroni multiple-comparisons testing was conducted to test for the effect of eye position. The statistical significance level was set to <5%. The statistical analysis software, IBM SPSS Statistics for Windows, ver. 20 (IBM Corp., Armonk, NY), was used.

## Results

**Figure 2:**
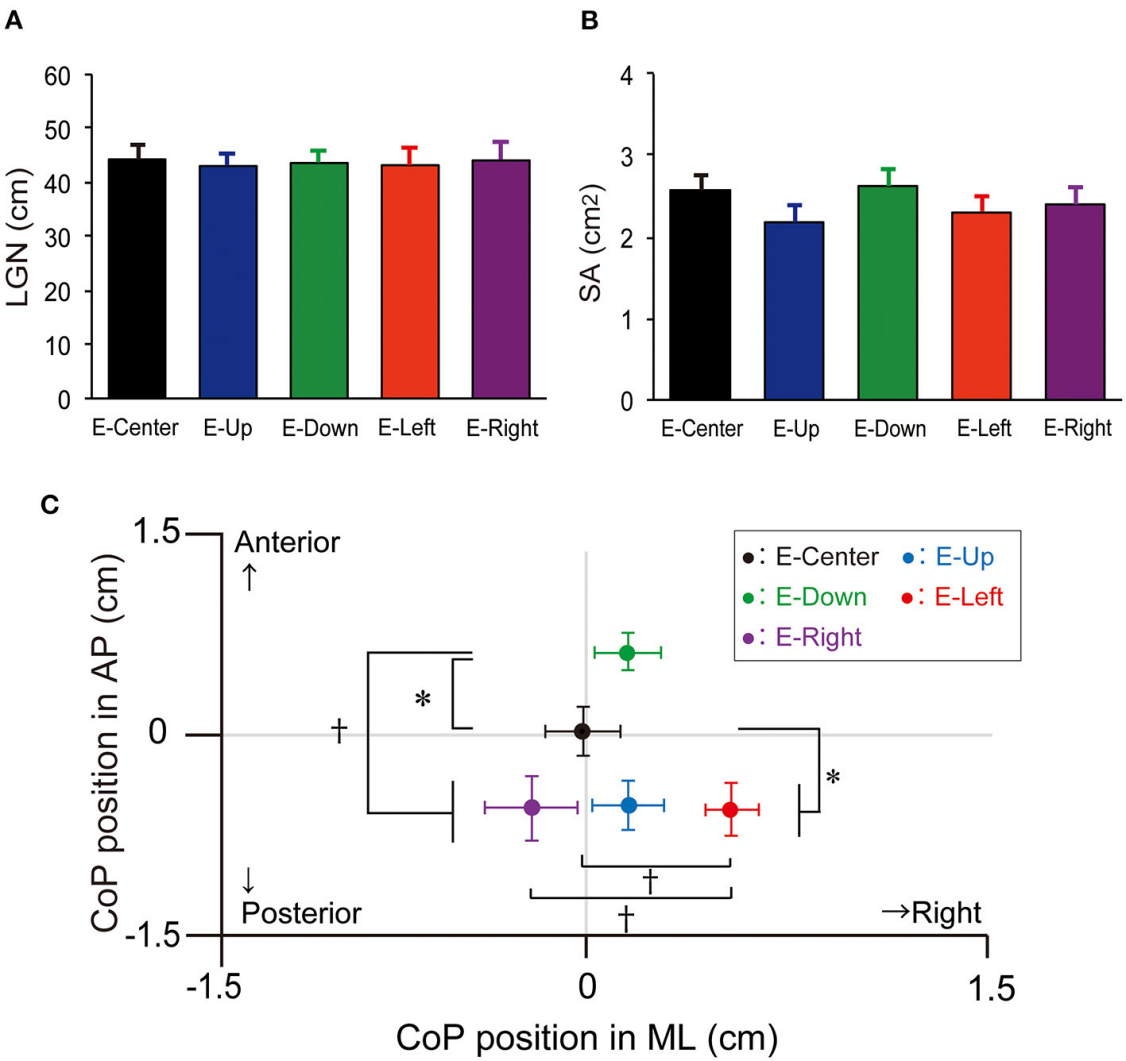
LNG, SA, and mean position of the CoP. **(A)** LNG and **(B)** SA with the E-Up, E-Down, E-Right, E-Right, and E-Center eye positions. The vertical bars reflect the mean LNG and SA. The error bars indicate the standard error of the mean. **(C)** The mean CoP position in the ML and AP directions. The solid circles indicate the mean CoP position. The error bars indicate the standard error of the mean. *p < 0.05, ^†^*p* < 0.01. LNG, total trajectory length; SA, sway area; CoP, center of pressure; ML, mediolateral; AP, anteroposterior.

The one-way ANOVA revealed that the main effect on LNG was not significantly different [*F*_(4, 34)_ = 0.03, *p* = 0.998, effect size (η^2^) = 0.001]. The LNG in each eye position was as follows: E-Center, 44.2 ± 2.9 cm (mean ± standard error of the mean); E-Up, 43.1 ± 2.6 cm; E-Down; 43.6 ± 2.5 cm; E-Right, 43.2 ± 3.3 cm; and E-Left, (44.1 ± 3.3 cm), with no significant difference in any position (Table 1; Figure 2A). The results of *post-hoc* testing between each group are summarized in Table 2.

**Table 1:** Results of CoP sway measurements.

	**E-Center**	**E-Up**	**E-Down**	**E-Right**	**E-Left**
LNG (cm)	44.2 ± 2.9	43.1 ± 2.6	43.6 ± 2.5	43.2 ± 3.3	44.1 ± 3.3
SA (cm^2^)	2.6 ± 0.2	2.2 ± 0.2	2.6 ± 0.2	2.3 ± 0.2	2.4 ± 0.2
Mean AP direction (cm)	0.0 ± 0.2	−0.6 ± 0.2	0.7 ± 0.1	−0.6 ± 0.2	−0.6 ± 0.3
Mean ML direction (cm)	−0.0 ± 0.1	0.2 ± 0.1	0.2 ± 0.1	0.5 ± 0.1	−0.2 ± 0.2

*Data are presented as mean ± standard error of the mean. CoP, center of pressure; LNG, total trajectory length; SA, sway area; Mean AP, mean of the CoP position in the anteroposterior direction; mean ML direction, mean of the CoP position in the mediolateral direction*.

**Table 2:** Results of *post-hoc* testing.

		**Eye position**	***p*** **-value**			
			**LNG**	**SA**	**AP**	**ML**
E-Center	vs.	E-Up	>0.99	>0.99	0.046	>0.99
		E-Down	>0.99	>0.99	0.023	>0.99
		E-Right	>0.99	>0.99	0.032	0.068
		E-Left	>0.99	>0.99	0.036	0.034
E-Up	vs.	E-Down	>0.99	>0.99	<0.001	>0.99
		E-Right	>0.99	>0.99	>0.99	0.607
		E-Left	>0.99	>0.99	>0.99	0.743
E-Down	vs.	E-Right	>0.99	>0.99	<0.001	0.574
		E-Left	>0.99	>0.99	<0.001	0.783
E-Right	vs.	E-Left	>0.99	>0.99	>0.99	0.003

*p-values were calculated with Bonferroni correction. LNG, total trajectory length; SA, sway area; Mean AP, mean of the CoP position in the anteroposterior direction; mean ML direction, mean of the CoP position in the mediolateral direction*.

The one-way ANOVA revealed that the main effect of SA was not significantly different [*F*_(4, 34)_ = 0.764, *p* = 0.550, effect size (η^2^) = 0.02]. The SA in each eye position was as follows: E-Center, 2.6 ± 0.2 cm^2^; E-Up, 2.2 ± 0.2 cm^2^; E-Down, 2.6 ± 0.2 cm^2^; E-Right, 2.3 ± 0.2 cm^2^; and E-Left, 2.4 ± 0.2 cm^2^, with no significant difference in any position (Table 1; Figure 2B). The results of *post-hoc* testing between each group are summarized in Table 2.

The one-way ANOVA revealed that the main effect of the mean CoP position in the AP direction was significantly different [*F*_(4, 34)_ = 7.267, *p* = 0.001, effect size (η^2^) = 0.16]. The mean CoP position in the AP direction in each eye position was as follows: E-Center, was 0.0 ± 0.2 cm; E-Up, −0.6 ± 0.2 cm; E-Down, 0.7 ± 0.1 cm; E-Right; −0.6 ± 0.2 cm; and E-Left, (−0.6 ± 0.3 cm). The mean CoP shifted significantly posteriorly in the E-Up, E-Left, and E-Right eye positions, and anteriorly in the E-Down eye position. A plus sign reflects an anterior shift, and a minus sign reflects a posterior shift (Table 1; Figure 2C). The results of *post-hoc* testing between each group are summarized in Table 2.

The one-way ANOVA revealed that the main effect of the mean CoP position in the ML direction was significantly different [*F*_(4, 34)_ = 3.714, *p* = 0.006, effect size (η^2^) = 0.08]. The mean CoP position in the ML direction was −0.0 ± 0.1 cm for E-Center, 0.2 ± 0.1 cm for E-Up, 0.2 ± 0.1 cm for E-Down, 0.5 ± 0.1 cm for E-Right, and −0.2 ± 0.2 cm for E-Left. The mean CoP shifted significantly to the right in the E-Right position, and to the left in the E-Left position. A plus sign reflects a shift to the right, and a minus sign reflects a shift to the left (Table 1; Figure 2C). The results of *post-hoc* testing between each group are summarized in Table 2.

## Discussion

We hypothesized that the body sway would be affected by the extraocular muscle proprioception due to eye position, even in the absence of visual references. The results showed that none of the eye positions had a significant effect on LNG or SA. However, eye fixation in the E-Down position shifted the CoP anteriorly, while those in the E-Right, E-Left, and E-Up positions shifted it posteriorly. The CoP was shifted to the left only with eye fixation in the E-Left position, but to the right in the E-Up, E-Down, and E-Right positions. As a result, eye position without visual information does not increase LNG or SA but seems to shift CoP as a form of postural control.

LNG and SA did not differ significantly for any eye position. LNG assesses the length of the CoP trajectory in terms of distance, while SA evaluates the size of the CoP area, indicating that both parameters reflect the degree of body sway during upright standing. A previous study demonstrated that the fixation of gaze to a target on the right or left side may increase body sway under open-eye conditions (5). The change in optical flow in the peripheral visual field increases CoP displacement during upright standing (3). Therefore, changes in visual reference may reflect a possible mechanism to increase body sway accompanied by gazing, and the lack of change in visual reference may underpin the lack of an effect of eye position on LNG and SA in this study. Further, changing the eye position in the absence of visual information may not increase the range of CoP swing. Several possible mechanisms underlie the shift in the CoP depending on eye position in the absence of visual information. The first is eye and body coordination for gazing, as the CoP shifts for the upper limb, lower limb, trunk, and head in anticipatory and compensatory directions to decrease body sway accompanied by limb movement (20) and to decrease retinal slip under open-eye conditions (21). When gazing at the foot while standing, the neck and trunk are bent to enable the process. In contrast, when looking up above the head, the neck and trunk are extended for gazing. In this study, the participants were asked not to move and to stand upright during the examination, but non-voluntary body movements may have occurred with different eye positions even though the eyes were closed. Therefore, the significant shift in the CoP to the anterior and posterior directions may reflect a coordinating movement of the body associated with non-voluntary gazing. Another possible mechanism, sensory feedback, is important for motor control. It reflects afferent sensory feedback from the extraocular muscles, as there was no visual feedback with the eyes closed during the examinations in this study. Only closing the eyes increases the CoP sway but does not shift the CoP in a specific direction (22). From the results of our study, it is interesting to note from our results that the CoP did not shift with the eyes in the E-Center position, but that all conditions except E-Left shifted the CoP to the right. Furthermore, only E-Down shifted the CoP anteriorly, while E-Up, E-Right, and E-Left shifted the CoP posteriorly. In other words, the eye position may induce a shift in the CoP in the direction of contraction of the external eye muscle. There is an interrelationship such that extraocular muscle proprioception affects the perception of body space and exterior space (23). Pettorossi et al. (24) stated that eye position contributes to movement perception, which our study supports. The factor that causes these CoPs to shift to the right is considered to be the influence of the dominant foot side since the asymmetry of the dominance of the lower limb affects upright postural control (25). In all participants, the dominant foot (17), defined as the side naturally used to kick a ball, was the right lower limb. The dependency of postural control with eye shift on the dominant lower limb may increase in response to the restriction of visual information. Based on this finding, we can hypothesize that the motor command to the lower limb, trunk, or head may be generated in conjunction with the eye position command even when the eyes are closed. In addition, this motor command accompanied by that for the eyes is quite robust and may not be sufficiently canceled by simply closing the eyes. Further, changing the eye position for dynamic gazing (18) or postural control (2) may be useful when the eyes are closed. Further experiments are warranted to verify these hypotheses.

The clinical implication of this study is that the direction of the eyes affects the body sway even without visual information such as that from the external environment. This relationship is an important finding in balance evaluation and training for people with ocular motility disorders due to various diseases.

There are several limitations to this study. First, the subjects were instructed by the examiner to fix their eyes in the direction indicated as much as possible without moving their heads. Therefore, it is not clear whether the subjects moved their eyes to their maximum capacity and whether the head was completely motionless. Second, all CoPs were shifted to the right except when the eyes were directed to the right. This was thought to be caused by the shift to the side of the dominant foot, but future experiments including participants with left-footedness are needed to verify this hypothesis. Finally, the maximum range of the eye position was not controlled because it was set arbitrarily by the subjects.

In conclusion, we found that change in eye position in the absence of visual references can induce deviation in the CoP depending on the eye position, with the direction possibly influenced by foot-dominance bias. The results of this study showed that eye positions affect body movements even without visual information such as that from the external environment and that there is a relationship between the eye positions and the body sway.

## Data Availability Statement

The original contributions presented in the study are included in the article/Supplementary Material, further inquiries can be directed to the corresponding author.

## Ethics Statement

The studies involving human participants were reviewed and approved by Shijonawate Gakuen University, Faculty of Rehabilitation Research Ethics Committee (Approval No. 18-10). The patients/participants provided their written informed consent to participate in this study.

## Author Contributions

YT: conceptualization, funding acquisition, methodology, resources, software, supervision, validation, writing—original draft, and data curation. YT and AM: formal analysis, visualization, and writing—review and editing. Both authors contributed to the article and approved the submitted version.

## Funding

This study was supported by Grant Number IHSS2001 from the Shijonawate Gakuen University Health Science Research Support Fund.

## Conflict of Interest

The authors declare that the research was conducted in the absence of any commercial or financial relationships that could be construed as a potential conflict of interest.

## Publisher's Note

All claims expressed in this article are solely those of the authors and do not necessarily represent those of their affiliated organizations, or those of the publisher, the editors and the reviewers. Any product that may be evaluated in this article, or claim that may be made by its manufacturer, is not guaranteed or endorsed by the publisher.
